# Exploring the diversity of produced water bacteria with hydrocarbon-degrading potential using MALDI-TOF MS and multivariate statistical analyses

**DOI:** 10.1007/s10529-025-03641-0

**Published:** 2025-09-26

**Authors:** Maryam Al-Kaabi, Nabil Zouari, Mohammad Yousaf Ashfaq, Mohammad A. Al-Ghouti

**Affiliations:** Environmental Sciences Program, Department of Biological and Environmental Sciences, College of Arts and Sciences, Qatar University, P.O.B 2713, Doha, Qatar

**Keywords:** Hydrocarbon-degrading bacteria, Produced water, Bacterial diversity, MALDI-TOF MS, Enrichment cultures

## Abstract

The success of bioremediation of produced water relies on the use of hydrocarbon-degrading bacteria. Hence, the selection of highly tolerant endogenous strains from produced water is crucial to designing successful bioremediation. However, the employed isolation and screening approaches are, in general, long. Integrative and rapid approaches based on microbiological and molecular techniques are now required due to the frequent fluctuation of the composition of the produced water. Here, enrichment cultures at high toxicity followed by protein profiling using MALDI-TOF MS were shown to be efficient in clustering the endogenous hydrocarbon-degrading bacteria and help select the potential candidates. Several bacterial strains (*n* = 18) were isolated from produced water sampled from Qatar’s North Field natural gas production. Fourteen strains were identified as *Bacillus cereus* (*n* = 14), and one as *Staphylococcus hominis* (*n* = 1) using MALDI-TOF MS. Three strains were identified as *Aneurinibacillus humi* (*n* = 2) and *Aneurinibacillus aneurinilyticus* (*n* = 1) through ribotyping. The strains were further differentiated based on their protein profiles using MALDI-TOF MS and multivariate statistical analyses. Multivariate analyses (composite correlation index, principal component analysis, and dendrogram) demonstrated substantial diversity among the isolates, highlighting their potential as robust candidates for bioremediation and produced water treatment.

**Supplementary Information:**

The online version contains supplementary material available at 10.1007/s10529-025-03641-0.

## Introduction

One of the inevitable byproducts of oil wells is produced water, generated during the extraction process (Fakhru’l-Razi et al. 2009). It is frequently composed of complex mixtures of heavy metals, salts, hydrocarbons, and other pollutants that can harm both terrestrial and aquatic ecosystems if left untreated. The main oil components that may be present in produced water are light and heavy hydrocarbons, aromatics, asphaltenes, and hydrogen sulfides (Costa et al. 2022). Consequently, the remediation of the produced water requires processes such as separation, dehydration, and chemical treatment to remove hydrocarbons and reduce environmental impacts (Igunnu and Chen 2012; Jepsen et al. 2018). The advantage of bioremediation, which uses microorganisms, is the breakdown of hydrocarbons as a viable and sustainable process to treat produced water (Zhang et al. 2024). In fact, some bacteria have the capacity to break down hydrocarbons into less hazardous and simpler compounds, which helps clean up contaminated areas (Ezennubia and Vilcáez 2023). However, high salinity, fluctuations of hydrocarbon composition and mixtures, temperature, and inorganic components characterize produced waters (Ghosh and Chakraborty 2019). The occurrence and the capacity of hydrocarbon-degrading bacteria in produced water are highly affected by these conditions.

The microbial communities that may exist in produced water are characterized by their adaptation to such harsh conditions, which can lead to diversity of bacterial strains and degrading metabolic mechanisms. Application of non-native bacterial strains to remediate produced water leads to failure, in general (Christova et al. 2019). Indeed, native bacterial populations may develop symbiotic and competitive interactions, contributing to the overall bioactivity of hydrocarbon degradation (Cabrera et al. 2022). However, this type of community requires stable water composition to be sustained, which is not ensured during the treatment process (Olowomofe et al. 2019). Some of the native bacteria may be more active than others within these populations. They should exhibit a higher tolerance to toxicity and composition fluctuations and more suitable metabolic activities even at high hydrocarbon concentrations (Zhou et al. 2021). Isolation and selection of these native hydrocarbon-degrading bacterial strains is crucial to enhancing the effectiveness of any approach of bacterial bioremediation of produced water (Wang et al. 2018). Therefore, studying these populations of bacteria and their diversity is essential to simplify the approach of selection of candidates to enhance bioremediation for better effectiveness and rapidness. Cutting-edge methods like next-generation genomic sequencing and recent developments in microbiology (Bianconi et al. 2023) have made it possible to identify and characterize a wide variety of bacteria that break down hydrocarbons, some of which have special metabolic properties. These advancements allowed the selection and optimization of bacterial strains at special growth conditions. However, a more ecological and responsive approach to the study of the diversity of these bacteria is now necessary to demonstrate the diversity at the level of the protein content. Indeed, the consequence of the adaptation of each bacterial strain is translated into an adapted protein content, qualitatively and quantitatively, as a result of the expression of different genes or expression levels. The matrix-assisted laser desorption/ionization time-of-flight mass spectrometry (MALDI-TOF MS) method is a mass spectrometry technique used to identify and analyze bacteria by measuring the mass profiles of biomolecules, primarily proteins, present in the cells. The idea behind this technique is to ionize biological molecules using a laser that deposits energy in a matrix, allowing molecules to be ionized without significant fragmentation (Martina et al. 2018). These ions are then analyzed using a mass spectrometer based on their time of flight (TOF), resulting in a distinct picture profile that represents various biomolecules. MALDI-TOF MS is a simple method to generate a mass protein profile for each strain, thereby simplifying the analysis of similarities and differences compared to other isolates (Santos et al. 2016). It is well established for application in environmental microbiology (Martina et al. 2018; Santos et al. 2016). Multivariate statistical tools such as principal component analysis (PCA), hierarchical clustering (dendrogram), and composite correlation index (CCI) are powerful tools for interpreting complex protein fingerprints of microbes typically obtained through MALDI-TOF MS. The coupling of these techniques with MALDI-TOF MS enables discrimination of isolates at the genus, species, and strain levels and also allow diversity assessments across datasets (Pascale et al. 2024; Koster, and Brul 2016). The use of these tools can streamline the characterization of environmental bacteria in a fast and cost-effective manner through facilitating intuitive visualization of similarity patterns and enabling dereplication (Ashfaq et al. 2022, 2019).

In this study, the occurrence and diversity of hydrocarbon-degrading bacteria in produced water from Qatari gas wells are studied for the first time using MALDI-TOF MS. The main objective is to demonstrate the high diversity of hydrocarbon-degrading bacteria in produced water through multivariate statistical analyses and to guide the selection of representative strains of categories of the isolated native hydrocarbon-degrading bacteria for bioremediation. As a first step, the strains are isolated by a strategy based on enrichment cultures during which high toxicity pressure is applied using high concentrations of diesel hydrocarbons. The findings propose a rapid and efficient approach to selecting the appropriate strains for successful bioremediation approaches and reducing failures due to the limited adaptability of the hydrocarbon-degrading bacteria used. This approach will be useful in the screening approaches of isolates for the bioremediation of produced water by selecting representatives of the differentiated categories.

## Material and methods

### Samples collection

A regular sampling procedure was followed to guarantee the integrity and representativeness of the produced water samples from Qatar’s North Field natural gas production. Sterile and clean glass sample containers, free of inorganic and organic pollutants, were used. Before sample collection, the sampling line was flushed to get rid of any debris or standing water. The generated water was collected straight into the ready-made containers using an automatic sampler. To avoid losing volatile components, the sample was collected with as little exposure to air as possible.

### Isolation of hydrocarbon-degrading bacteria by enrichment cultures

The hydrocarbon-degrading bacteria were isolated from the produced water by an enrichment culture procedure, using the mineral salt medium (MSM) as described by Al-Kaabi et al. (2020) with modifications. A mixture of 12 mL of sterile MSM was mixed aseptically with 3 mL of produced water and incubated at 30 °C for 3 d. Then, 3 mL were used to inoculate 16 mL MSM supplemented with 1 mL diesel. The 5% (v/v) diesel corresponds to 37.5 g/L diesel hydrocarbons. The diesel (ρ = 0.750 kg/L), used in this study, was kindly provided by QE oil refinery Unit (UmSaeed, Qatar). After three successive cultures, 100 µL were spread on MSM solid plates coated with 100 µL diesel. The growing colonies were separately picked up in LB plates, then purified by five successive subculturing. The strains that were able to grow on solid MSM plates coated with oil within 48 h were considered in this study. The purified isolates were preserved at − 20 °C in LB supplemented with 30% glycerol.

### Identification of the hydrocarbon-degrading bacterial isolates using MALDI-TOF MS

The ethanol/formic acid extraction method was used as recommended by the manufacturer and widely reported in the literature (Ashfaq et al. 2019). In the first step, a loop full of bacterial cells from an overnight-grown culture in MSM-oil solid medium was suspended in 300 µL of autoclaved distilled water. 900 µL of ethanol was then added to it, followed by thorough mixing and then centrifugation at 13,000 rpm for 2 min. The supernatant was discarded, and the pellet was again re-suspended in the mixture of formic acid (70%) and acetonitrile (100%) at a 1:1 (v/v). After centrifugation (13,000 rpm, 2 min), the supernatant was then collected, as extracted bacterial proteins were used for the identification step. The identification was carried out using a MALDI-TOF MS instrument (Model: microflex LT/SH from Bruker Daltonics, Germany). The extracted bacterial proteins (1 µL) were first deposited on the MALDI biotarget plate. For each strain, two technical replicates were used. After air drying, the deposited proteins were overlaid with α-cyanohydroxycinnamic acid (CHCA) matrix for crystallization. Once dried, the identification process was initiated by loading the biotarget plate into the machine and after labelling each spot in Biotyper Real Time Classification software. A log scale with a score ranging from 0 to 3 was used to represent the profile matching. The highest score obtained for each strain was reported. According to the manufacturer’s instructions, the value was interpreted as follows: a score of 2.3–3.000 indicates a highly probable species-level identification, a score of 2.00–2.299 indicates genus identification and probable species-level identification, and a score of 1.70–1.999 indicates a probable genus-level identification. Bacterial Test Standards (Bruker Daltonics, Bremen, Germany; item catalog #255343) were used for the calibration.

### Acquisition of protein mass spectra of isolates through MALDI-TOF MS

The Bruker flex control was used to acquire protein mass spectra. The laser frequency was 60 Hz, and the laser intensity was 35% with 20 and 18.7 kV of acceleration and source voltages, respectively. 240 shots of laser beam in 40-shot steps on different areas of the sample were gathered for each mass spectrum. Since there were two spots for each sample, and each sample was run three times, a total of six MS spectra for each strain were thus acquired.

The raw spectral data obtained from three replicates were pre-processed using Flex Analysis software. This pre-processing involved smoothing, baseline subtraction, and peak identification using default algorithms, followed by a thorough quality assessment of each spectrum. Spectra exhibiting poor quality—characterized by high background noise or abnormal intensity values—were excluded from further processing. The remaining high-quality spectra were then used to generate Main Spectra Projections (MSPs) through the automated MSP creation function in the Bruker Biotyper Compass software. Each MSP encapsulated information on the mean peak masses, mean intensities, and mean frequencies. A total of 18 MSPs, each corresponding to a distinct strain, were subsequently subjected to multivariate statistical analyses.

### Multivariate statistical analyses of protein mass spectra of isolates

The multivariate statistical tools used for the analyses of mass spectra were principal component analysis (PCA), hierarchical clustering (dendrogram), and composite correlation index (CCI). For PCA, the Bio Typer PCA Clustering Standard Method was used. The method of clustering was chosen as hierarchical, the distance measure as correlation, and the linkage algorithm was set as average. The mass upper bound and lower bound were set as 15,000 and 3000 m/z, respectively, with a resolution of 2 m/z. Similarly, Bio Typer MSP Dendrogram Creation Standard Method was used for dendrogram analysis, where the distance measure was selected as correlation, and linkage as average. PCA and dendrogram helped to differentiate the strains based on their differences in the protein mass spectrum. The closely related strains were clustered together. Each cluster was then plotted in a 3D PCA graph, demonstrating the difference between each cluster.

To calculate the CCI for isolates, the raw spectral data were first organized into m/z bins with a class interval of 5 m/z, ensuring that peaks across spectra could be compared within standardized mass-to-charge ranges (Kehrmann et al. 2016; Ashfaq et al. 2019). Each spectrum was then normalized to account for differences in overall intensity and to make the datasets directly comparable. Using these normalized values, the CCI was computed in Excel by generating pairwise correlation coefficients between the binned intensity profiles of all spectra. The correlation coefficient (*r*) ranges from 0 to 1, where a value of 0 indicates no similarity (spectra are completely uncorrelated) and values close to 1 indicate very high similarity (spectra are highly correlated). The overall CCI was obtained as the average of these pairwise correlation values, providing a quantitative measure of overall diversity across the dataset.

### Identification of the bacterial isolates by ribotyping

Cells were grown overnight at 30 °C on LB plates, and then their DNA was extracted. After being suspended in 0.5 mL of distilled water, the cells were heated for 10 min in a water bath and centrifuged at 12,300 g for 10 min. A fresh PCR amplification tube was filled with the supernatant, which contained whole DNA. Universal primers RibS73sp 5′-AGAGTTTGATCCTGGCTCA-3′ and RibS74sp 5′-AAGGAGGTGATCCAGCCGCA-3′ were used to amplify the bacterial strains’ 16S rRNA gene segments (1500 bp). A total of 25 μL of PCR buffer containing 1.5 μM MgCl_2_, 0.8 μM dNTP, 1.35 μM of both forward and reverse primers, 10–20 ng of the isolates’ genomic DNA utilized as a template for PCR reactions, and 0.5 IU Taq DNA polymerase was used for each PCR reaction. Each PCR reaction’s thermocycler schedule started with a three-minute initial denaturation step at 94 °C. 35 cycles of 45-s denaturation steps at 94 °C, 45-s annealing steps at 50 °C, and 45-s elongation steps at 72 °C were then followed by a final 2-min extension step at 72 °C. The QIAquick PCR Purification Kit from Qiagen was used to purify the amplicons. The purified amplicons were subjected to a secondary PCR, and the Applied Biosystems BigDye X-Terminator Purification Kit was used for the last purification procedures. An Applied Biosystems 3500 Series Genetic Analyzer was used for the sequencing process. The NCBI Blast server was used to identify the most closely similar sequence among those found in the GenBank database based on the 16S rRNA gene sequences that were received from each isolate.

## Results and discussion

### Isolation and identification of hydrocarbon-degrading bacterial strains from the produced water

The process of isolating endogenous hydrocarbon-degrading bacteria from the produced water samples from the oil fields was performed through the enrichment culture approach with multiple steps. The objective was to focus on the bacterial isolates that tolerate high toxicity exhibited by the hydrocarbons from the produced water, then from diesel used at 5% (corresponding to 37.5 g/L). Following several cycles of inoculation of fresh cultures and incubations, the cultures were enriched with the most tolerant strains, which were isolated and purified. The strains that were able to grow on solid MSM plates coated with oil were considered in this study (Table 1). The purified strains were identified using the MALDI-TOF MS technique, which generates an average spectrum for each strain. Complex collections of discrete ions with m/z ratios ranging from 2000 to 10,000 were revealed by the mass spectra. The generated peak intensity enables the detection of unique proteins, which are biomarkers among closely related isolates. As a result, the indicated peaks are identified as genus-specific biomarkers for the genera known in the database. Moreover, at the species level, the mass spectra can appear highly similar. This indicates that these protein peaks could potentially serve as species-specific biomarkers. Table 1 lists the isolated bacterial strains from the Qatari north field PW samples and their identification using MALDI-TOF MS. Interestingly, 14 isolates were identified at the genus and species level as *Bacillus cereus*, and one as *Staphylococcus hominis*.

**Table 1:** List of isolated hydrocarbon-degrading bacterial strains from the North Field produced water and their identification using MALDI-TOF MS

Strain Code	Identification by MALDI TOF	MALDI Score	Reliability of identification
SBS1	*Bacillus cereus*	2.36	Highly probable at species level
SBS2	*Bacillus cereus*	2.28	Probable at species level and accurate at genus level
MK1	*Bacillus cereus*	2.17	Probable at species level and accurate at genus level
MK2	*Staphylococcus hominis*	2.12	Probable at species level and accurate at genus level
MK4	*Bacillus cereus*	2.14	Probable at species level and accurate at genus level
MK5	*Bacillus cereus*	2.21	Probable at species level and accurate at genus level
MK6	*Bacillus cereus*	2.14	Probable at species level and accurate at genus level
MK7	*Bacillus cereus*	2.25	Probable at species level and accurate at genus level
MK9-1	*Bacillus cereus*	2.22	Probable at species level and accurate at genus level
MK9-2	*Bacillus cereus*	2.22	Probable at species level and accurate at genus level
MK10	*Bacillus cereus*	2.17	Probable at species level and accurate at genus level
MK11-1	*Bacillus cereus*	2.11	Probable at species level and accurate at genus level
MK11-2	*Bacillus cereus*	2.16	Probable at species level and accurate at genus level
MK12	*Bacillus cereus*	2.22	Probable at species level and accurate at genus level
MK14	*Bacillus cereus*	2.32	Highly probable at species level

However, three isolates (MK3, MK8, and MK13) could not be identified by MALDI-TOF MS, possibly due to the lack of or missing reference protein spectra in the database for this genus. The three isolates were identified by ribotyping. The isolates MK8 and MK3 were identified as *Aneurinibacillus humi* and the strain MK13 was identified as *Aneurinibacillus aneurinilyticus*. Their corresponding GenBank Accession Numbers are mentioned in Table 2.

**Table 2:** Identification of three hydrocarbon-degrading bacterial strains from the North Field produced water and their identification by ribotyping:

Strain Code	Code	Identity	Similarity %	Genebank Accession No	Code	Identity	Similarity %	Genebank Accession No
MK8	17F	*Aneurinibacillus humi*	97.37	NR_169383.1	17R	*Aneurinibacillus aneurinilyticus*	96.32	ON243745.1
MK3	19F	*Aneurinibacillus humi*	97.22	NR_169383.1	19R	*Aneurinibacillus aneurinilyticus*	95.84	ON243745.1
MK13	21F	*Aneurinibacillus aneurinilyticus*	96.21	ON243745.1	21R	*Aneurinibacillus aneurinilyticus*	96.21	ON243745.1

*Bacillus cereus* (*B. cereus*) is the most dominant bacterium in the produced water, as high tolerant to the high hydrocarbon concentration employed in the isolation procedure. It is known for its ability to degrade petroleum hydrocarbons and different organic pollutants, with numerous reports showing its presence at oily-contaminated sites (Al Disi et al. 2017; Al Kaabi et al. 2022; Eldos et al. 2024). Its proficiency in degrading petroleum hydrocarbons and various organic contaminants renders it an important microorganism for bioremediation initiatives in polluted environments (Al Kaabi et al. 2022). Indeed, *B. cereus* is recognized for its versatile metabolism that can degrade different hydrocarbons for carbon and energy sources (Al Sayegh et al. 2021). Strains of *B. cereus* had been known to degrade alkanes, aromatic compounds, and petroleum hydrocarbons. This bacterium produces a variety of enzymes, like alkane monooxygenases and aromatic-ring hydroxylating dioxygenases, which allow the bacterium to degrade hydrocarbons to simpler forms that can be degraded and metabolized. Being able to form spores makes surviving in high-hydrocarbon and low-oxygen areas easier, but it is still challenging to grow. It had been reported significant potential of *B. cereus* for large-scale bioremediation initiatives designed to restore oil-contaminated ecosystems (Kebede et al. 2021). *Staphylococcus hominis*, which was recently reported as a biosurfactant producer using oil hydrocarbons, has shown its presence in hydrocarbon-polluted sites, which indicates some possible metabolic capability to tolerate or degrade these compounds (Al Marri et al. 2023). This bacterium is mostly found on human skin and other microbiological environments, but may be located in certain contaminated areas, where it could play a part in breaking down oil spills. The strains identified by ribotyping belong to *Aneurinibacillus,* with two of them belonging to the species *humi*, and one to the species *aneurinilyticus*. *Aneurinibacillus* is classified within the *Bacillaceae* family.

The strain MK8 was identified as *Aneurinibacillus humi* with 97.37% similarity and as *Aneurinibacillus aneurinilyticus* with 96.32% similarity. The strain MK3 was identified as *Aneurinibacillus humi* with 97.22% similarity and as *Aneurinibacillus aneurinilyticus* with 95.84% similarity. However, the strain MK13 was uniquely identified as *Aneurinibacillus aneurinilyticus* with 96.21% similarity. The ribotyping results confirmed that all strains are within the genus *Aneurinibacillus* with some degree of closeness to other species, which denotes their genetic relationship.

*Aneurinibacillus* is well-known for its ability to break down complex organic substances, and it may be useful in bioremediation, specifically in the hydrocarbon degradation process (Palanimanickam and Sepperumal 2017). In addition, this bacterium has specific enzymes involved in the hydrocarbon-degrading process of petroleum, and those capable of degrading petroleum hydrocarbons, mainly alkanes and aromatic compounds (Palanimanickam and Sepperumal 2017; Lee and Lee 2016). Regardless, the efficiency of hydrocarbon degradation, as well as its role in bioremediation, is not as well defined as for other extensively studied genera like *Pseudomonas or Bacillus*. However, our findings clearly showed that the isolated strains of *Aneurinibacillus* are highly tolerant to high hydrocarbon concentrations, which has never been reported before.

### Study of the protein’s profiles of the isolated hydrocarbon-degrading bacterial strains

**Fig. 1:**
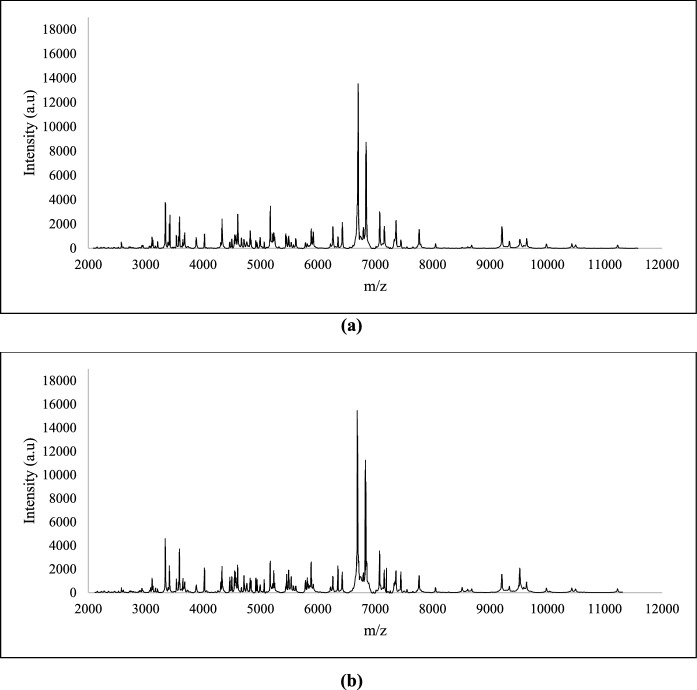
MALDI-TOF protein spectra of the isolates **a** SBS 1 and **b** SBS 2 identified as *B. cereus*

**Fig. 2:**
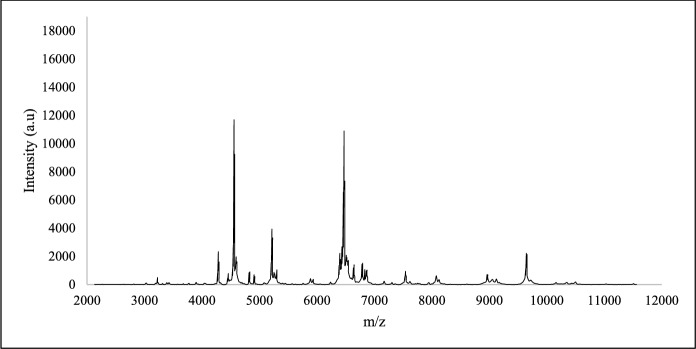
MALDI-TOF protein spectrum of the MK2 identified as *S. hominis*

**Fig. 3:**
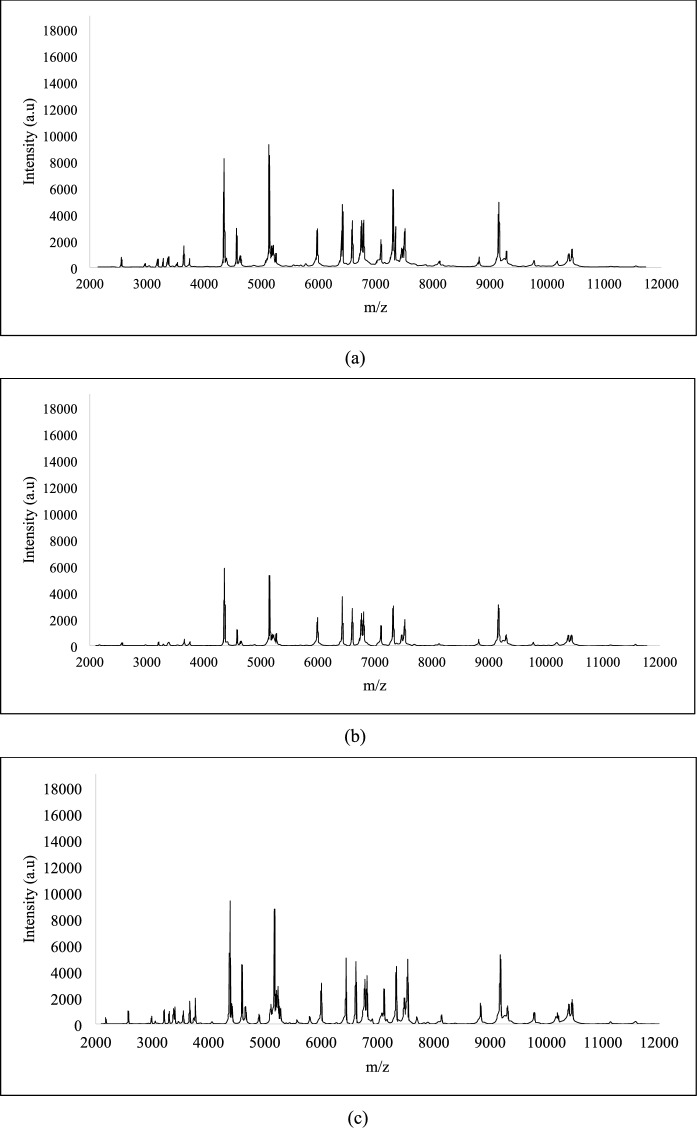
MALDI-TOF protein spectra of the unidentified isolates **a** MK3, **b** MK8, and **c** MK13

The process of isolating the hydrocarbon-degrading bacterial strains from the produced water was performed using enrichment cultures, which could lead to the isolation of the same strain more than once. The identification using MALDI-TOF MS or ribotyping is not appropriate to differentiate between them. The unique protein profile for each isolate, as generated by MALDI-TOF MS, was employed for their differentiation. The protein profile of each strain is formed by the proteins with m/z values from 2000 to 20,000. As shown in Fig. 1, the profiles of the strains SBS1 and SBS2, identified as *B. cereus*, indicate some similarities and possible differences in their proteomic composition. Profile of MK2 (identified as *S. hominis*) shows a unique protein profile, as the peaks vary in intensity and position compared to SBS1 and SBS2 (Fig. 2). Similarly, the protein profiles of isolates belonging to *Aneurinibacillus,* obtained through MALDI-TOF MS and presented in Fig. 3, mark clear differences as compared to the profiles of *B. cereus* (Fig. 1) and *S. hominis* (Fig. 2). The apparent differences between the profiles (Figs. 1, 2, and 3) highlights how bacteria belonging to different genera express proteins of variable m/z values.

**Fig. 4:**
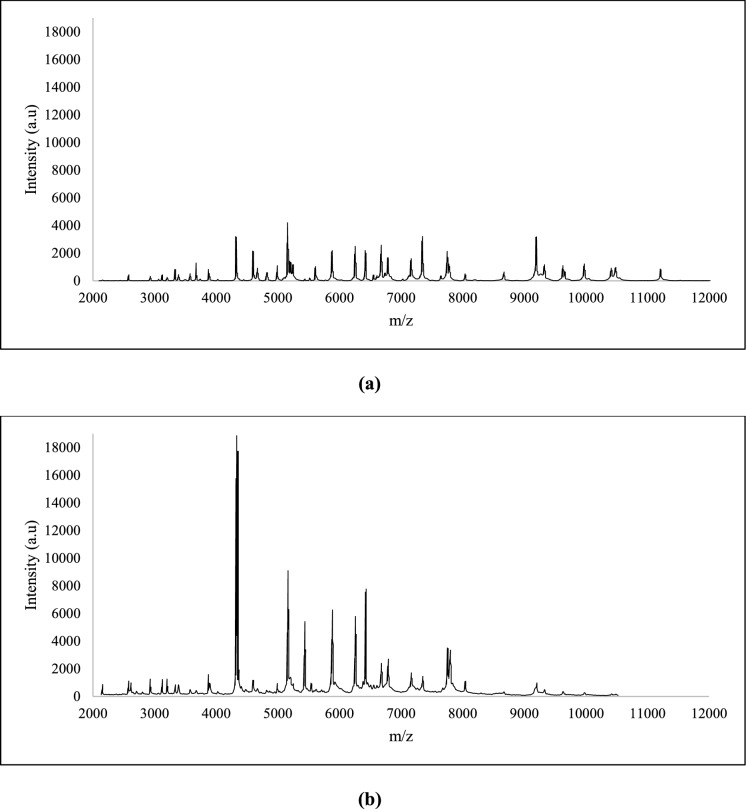
MALDI-TOF protein spectra of the isolates **a**) MK11-1 and **b** MK14, highlighting the differences between them, although both were identified as *B. cereus*

The observed protein profiles of all the other isolates exhibit distinct peaks characteristic of their protein signatures, which allows for their differentiation. In this context, as depicted in Figure S1, MK1 and MK4 possess distinct characteristics in the 3000–4000 m/z range, whereas MK5, MK6, and MK7 display a shared signature in the 5000–6000 m/z range. The strains MK9-1 and MK9-2 exhibit very similar profiles, indicating a strong connection. MK10 is notable for its high-intensity spikes in the 7000–8000 m/z range. MK12 showcases unique peaks around 3500 and in the 6000–6500 m/z range that are absent in the other strains (Fig. S1). MK 11–1, and MK14 features distinct peaks in the 8000–9000 m/z range as shown in Fig. 4, indicating differences in their protein profiles.

The protein mass spectra of various bacterial strains exhibit clear differences in peak intensities, indicating variations in the levels of specific proteins. Even though these observations might suggest differential expressions, the assumptions regarding peak independence and the significant risk of drawing incorrect conclusions from multiple comparisons do not support the use of univariate statistical analysis methods to accurately discuss the differences between the strains, given that MALDI-TOF spectra are fundamentally high-dimensional. Instead, multivariate methods like CCI and PCA were demonstrated as suitable. PCA, an ordination technique rather than a statistical method, is utilized to visualize overall patterns and possible clustering among isolates according to their MALDI-TOF MS profiles and functional traits. PCA and hierarchical clustering (dendrograms) offer an overall perspective of spectral variance and more accurately represent biological connections between strains. These unsupervised methods capture the complete proteomic profile of every strain and are commonly endorsed for this kind of exploratory analysis. Therefore, multivariate statistical analyses were conducted for the MALDI-TOF MS data, and the results are presented in the preceding sections.

### Study of the diversity of the isolated strains based on their protein contents

**Fig. 5:**
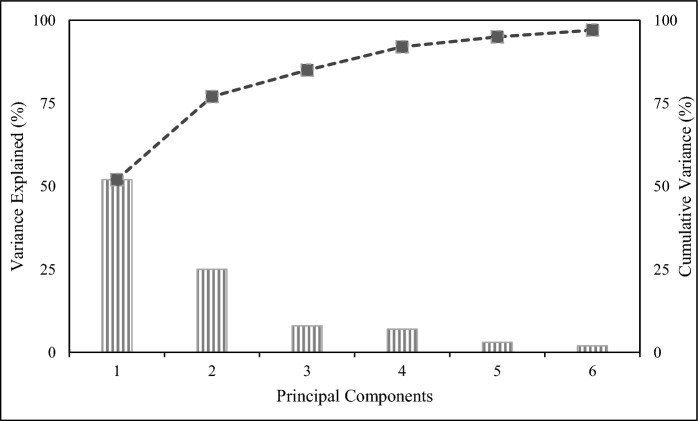
Classification of the studied strains using the percentage of variance explained

**Fig. 6:**
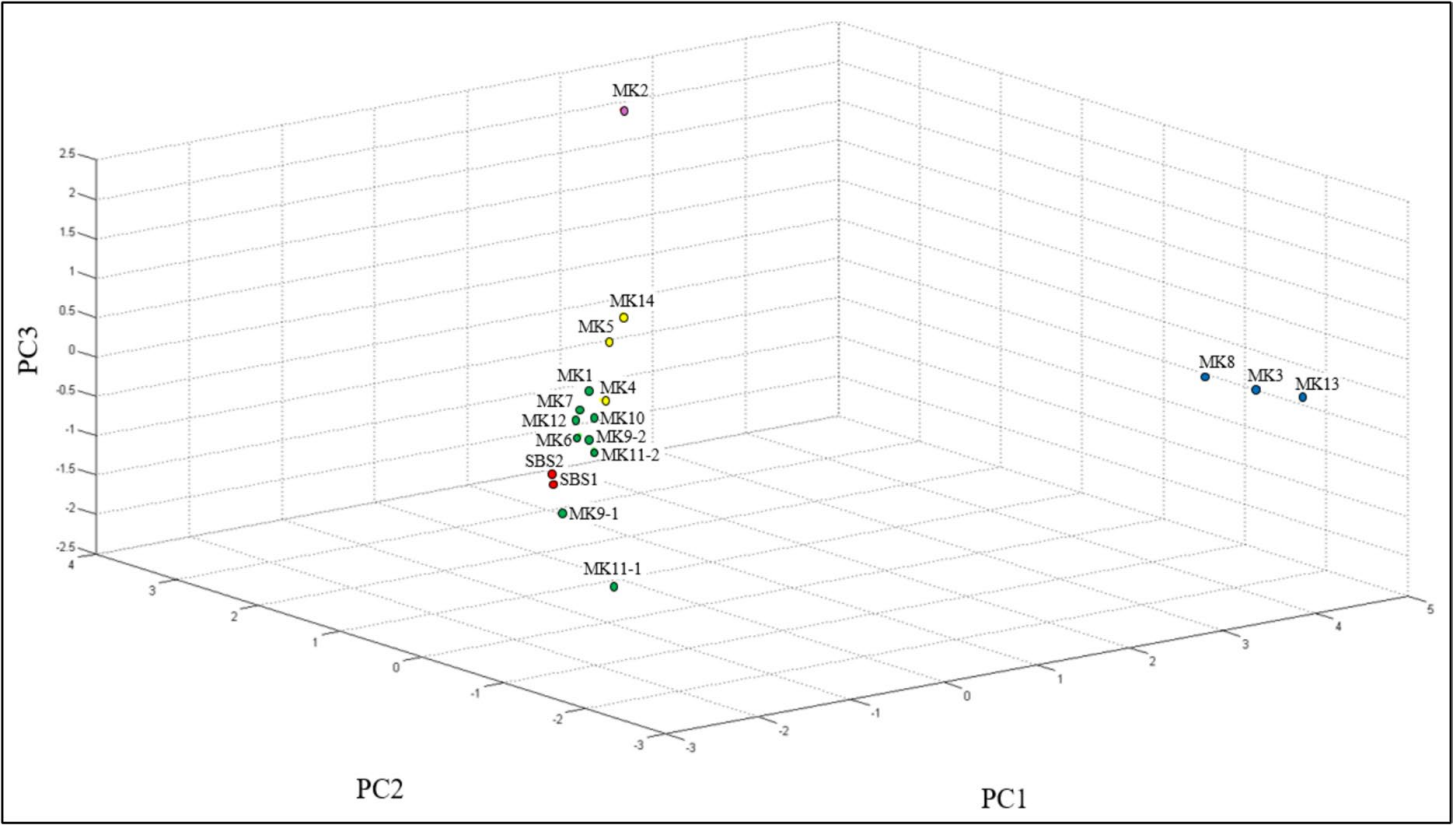
Classification of the studied strains using PCA clustering

**Fig. 7:**
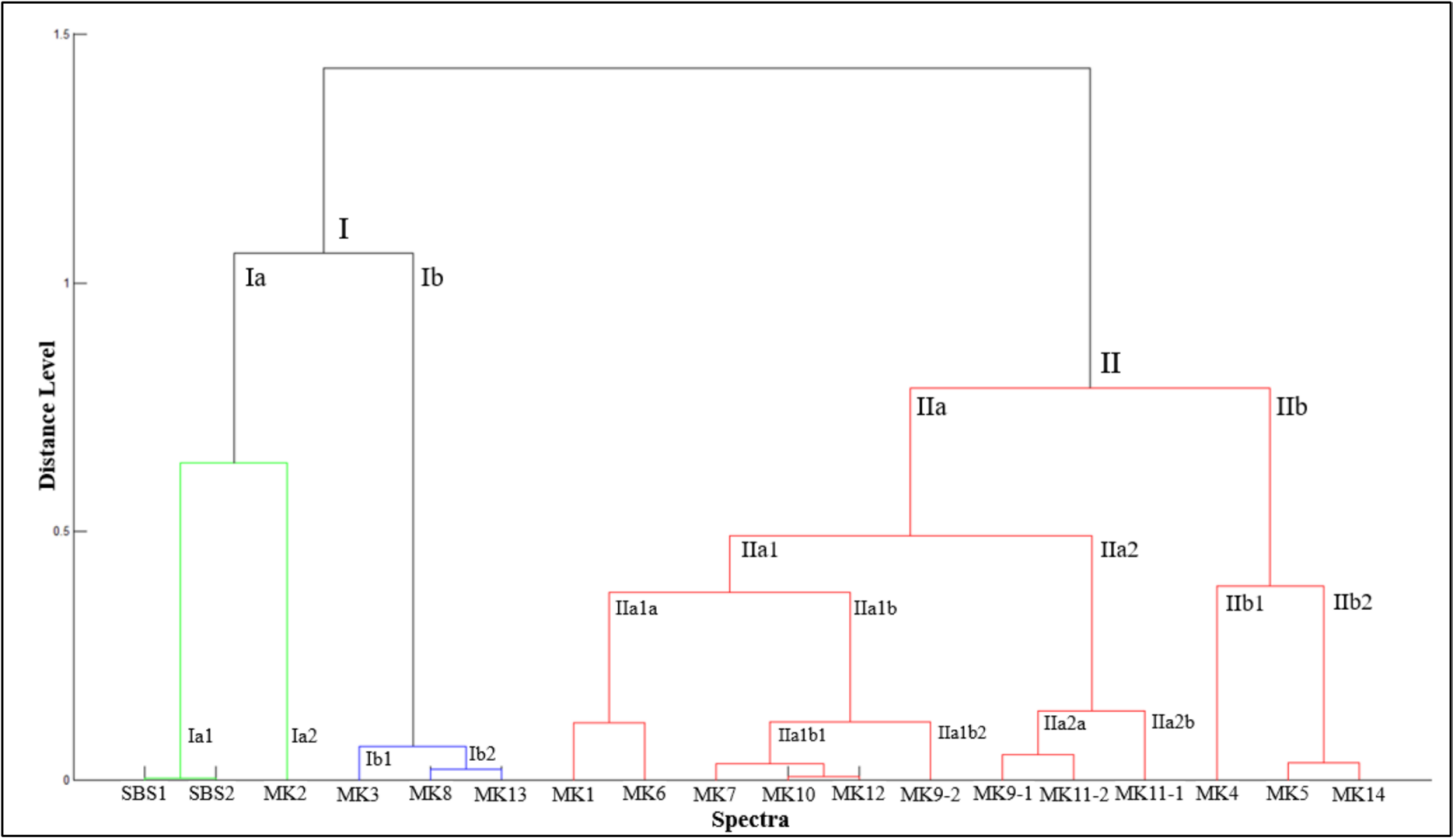
Dendrogram of the studied strains based on the protein profiles

The diversity of the potential hydrocarbon-degrading bacteria isolates from produced water was studied through multiple statistical approaches, namely the composite correlation index (CCI), hierarchical clustering (dendrogram), and principal component analysis (PCA). PCA was applied to reduce the dimensionality of the spectral data. As shown in Fig. 5, the three principal components, PC1 (52%), PC2 (25%), and PC3 (8%), collectively accounted for 85% of the total variance, which is considered sufficient for this type of study. The results of CCI are provided in Table 3, while the PCA 3D plot and dendrogram are shown in Figs. 6 and 7, respectively. The overall CCI value was 0.253, indicating high diversity among the bacterial isolates. Pearson r values in Table 3 are color-coded such that green color shows high similarity, yellow depicts moderate similarity, and red shows no similarity between the two spectra.

**Table 3:**
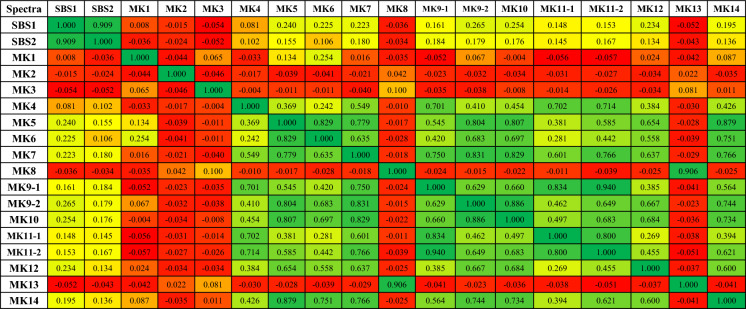
The CCI matrix showing pairwise spectral similarities among the 18 bacterial isolates

The spectra of SBS1 and SBS2 (both identified as *B. cereus*) exhibited high similarity with an *r* value of 0.909. This explains their clustering together in PCA (red cluster, Fig. 6) and their placement in the same subclade (Ia1) of the dendrogram (Fig. 7). In contrast, SBS1 and SBS2 showed negative *r* values against *S. hominis* (MK2) and *Aneurinibacillus* spp. (MK3, MK8, MK13), reflecting substantial differences across genera. Nevertheless, SBS1 and SBS2 were grouped in clade Ia under cluster I due to their shared dissimilarities (represented by moderate correlations) with other *B. cereus* strains in cluster II (MK5, MK6, MK7, MK9-1, MK9-2, MK11-1, MK11-2, MK12, MK14). Among *Aneurinibacillus* isolates, MK8 and MK13 were highly similar (*r* = 0.906), while MK3 correlated weakly with both (0.100 and 0.081, respectively) (Table 3). Despite this, their shared dissimilarity with other genera (negative *r* values) resulted in their grouping into clade Ib under cluster I in the dendrogram (Fig. 7) and their classification together as the blue cluster in PCA (Fig. 6).

MK2 showed slightly positive correlations with MK8 (0.042) and MK13 (0.022), but predominantly weak or negative correlations with most other isolates (MK1, MK4–MK7, MK9-1–MK12, MK14). This pattern explains its isolated position in PCA (purple category, distant from other colored clusters in Fig. 6) and its unique branching in cluster I of the dendrogram (Fig. 7).

The strains MK5, MK6, MK7, MK9-1, MK9-2, MK10, MK11-1, MK11-2, MK12, and MK14 showed high inter-correlations (~ 0.55–0.94), indicating similar protein fingerprints (Table 3). In contrast, MK1 correlated weakly, with its highest similarity observed with MK6 (*r* = 0.254), followed by MK5 (0.134) and MK14 (0.087). Interestingly, MK1 clustered with MK6 in the dendrogram, while MK5 and MK14 grouped with MK4 in a separate clade (Fig. 7). In PCA, MK1 was placed in the green category, whereas MK4, MK5, and MK14 were grouped as black circles (Fig. 6). The inclusion of MK4 in this group can be explained by its moderate correlation values with MK5 and MK14 (Table 3).

In summary, the combined results from PCA, dendrogram clustering, and CCI provide complementary evidence of substantial bacterial diversity among isolates. Statistically, the three methods showed strong agreement: closely related strains such as *B. cereus* (SBS1 and SBS2) and *Aneurinibacillus* (MK8 and MK13) consistently formed tight clusters, while MK2 showed weak correlations and appeared as an outlier in both PCA and the dendrogram. Biologically, these patterns reflect the coexistence of both highly similar protein fingerprints within the same genus and pronounced differences across genera, underscoring the presence of distinct hydrocarbon-degrading lineages in produced water. Together, these findings highlight not only the reliability of the statistical approaches but also the ecological significance of the observed diversity for hydrocarbon degradation potential.

## Conclusion

The diversity of hydrocarbon-degrading bacteria in produced waters is emphasized, and 18 strains were isolated and characterized. The predominant presence of *B. cereus* as compared to other strains called attention to the potential of these strains for bioremediation, by virtue of their metabolic adaptability and efficacy in degrading different hydrocarbons.

The combined statistical analyses (CCI, PCA, and dendrogram) revealed substantial diversity among the hydrocarbon-degrading bacterial isolates. While some strains within the same genus (e.g., *B. cereus*, *Aneurinibacillus*) showed high similarity, others, such as *S. hominis*, were markedly distinct, reflecting both intra-genus relatedness and inter-genus variability. This diversity highlights the presence of multiple lineages with potential roles in hydrocarbon degradation and supports the robustness of integrating complementary statistical approaches for microbial characterization. Importantly, such diversity may broaden the metabolic capabilities of microbial consortia, making these isolates promising candidates for applications in bioremediation and produced water treatment.

## Supplementary Information

Below is the link to the electronic supplementary material.

Supplementary file1 (DOCX 414 KB)

## References

[CR1] Al Disi Z, Jaoua S, Al-Thani D, Al-Meer S, Zouari N (2017) Considering the specific impact of harsh conditions and oil weathering on diversity, adaptation, and activity of hydrocarbon-degrading bacteria in strategies of bioremediation of harsh oily-polluted soils. Biomed Res Int 2017:8649350. 10.1155/2017/8649350 28243605 10.1155/2017/8649350 PMC5294359

[CR2] Al Kaabi N, Al-Ghouti MA, Jaoua S, Zouari N (2020) Potential for native hydrocarbon-degrading bacteria to remediate highly weathered oil-polluted soils in Qatar through self-purification and bioaugmentation in biopiles. Biotechnol Rep 28:e00543. 10.1016/j.btre.2020.e00543 10.1016/j.btre.2020.e00543 PMC7591726 33145191

[CR3] Al Kaabi N, Al Disi Z, Al-Ghouti M, Solling TI, Zouari N (2022) Interaction between indigenous hydrocarbon-degrading bacteria in reconstituted mixtures for remediation of weathered oil in soil. Biotechnol Rep 39:e00767. 10.1016/j.btre.2022.e00767 10.1016/j.btre.2022.e00767 PMC9562452 36245697

[CR4] Al Marri S, Eldos HI, Ashfaq MY, Saeed S, Skariah S, Varghese L, Mohamoud YA, Sultan AA, Raja MM (2023) Isolation, identification, and screening of biosurfactant-producing and hydrocarbon-degrading bacteria from oil and gas industrial waste. Biotechnol Rep 39:e00804. 10.1016/j.btre.2023.e00804 10.1016/j.btre.2023.e00804 PMC10300049 37388572

[CR5] Al Sayegh SY, Al Disi ZA, Al-Ghouti MA, Zouari N (2021) Evaluation by MALDI-ToF MS and PCA of the diversity of biosurfactants and their producing bacteria, as adaptation to weathered oil components. Biotechnol Rep 31:e00660. 10.1016/j.btre.2021.e00660 10.1016/j.btre.2021.e00660 PMC8446580 34557388

[CR6] Ashfaq MY, Al-Ghouti MA, Qiblawey H, Rodrigues DF, Hu Y, Zouari N (2019) Isolation, identification and biodiversity of antiscalant degrading seawater bacteria using MALDI-TOF-MS and multivariate analysis. Sci Total Environ 656:910–920. 10.1016/j.scitotenv.2018.11.477 30625677 10.1016/j.scitotenv.2018.11.477

[CR7] Ashfaq MY, Da’na DA, Al-Ghouti MA (2022) Application of MALDI-TOF MS for identification of environmental bacteria: a review. J Environ Manage 305:114359. 10.1016/j.jenvman.2021.114359 34959061 10.1016/j.jenvman.2021.114359

[CR8] Bianconi I, Aschbacher R, Pagani E (2023) Current uses and future perspectives of genomic technologies in clinical microbiology. Antibiotics 12(11):1580. 10.3390/antibiotics12111580 37998782 10.3390/antibiotics12111580 PMC10668849

[CR9] Cabrera J, Dai Y, Irfan M, Li Y, Gallo F, Zhang P, Liu X (2022) Novel continuous up-flow MFC for treatment of produced water: flow rate effect, microbial community, and flow simulation. Chemosphere 289:133186. 10.1016/j.chemosphere.2021.133186 34883132 10.1016/j.chemosphere.2021.133186

[CR10] Christova N, Kabaivanova L, Nacheva L, Petrov P, Stoineva I (2019) Biodegradation of crude oil hydrocarbons by a newly isolated biosurfactant producing strain. Biotechnol Biotechnol Equip 33(1):863–872. 10.1080/13102818.2019.1625725

[CR11] Costa TC, Hendges LT, Temochko B, Mazur LP, Marinho BA, Weschenfelder SE, de Souza SMGU (2022) Evaluation of the technical and environmental feasibility of adsorption process to remove water soluble organics from produced water: a review. J Petrol Sci Eng 208:109360. 10.1016/j.petrol.2021.109360

[CR12] Eldos HI, Zouari N, Saeed S, Ashfaq MYM, Al-Ghouti MA (2024) Isolation, identification, and characterization of potential biosurfactant-producing bacteria from processing wastewater for the development of eco-friendly green technology. Bioresour Technol Rep 25:101763. 10.1016/j.biteb.2024.101763

[CR13] Ezennubia V, Vilcáez J (2023) Removal of oil hydrocarbons from petroleum produced water by indigenous oil degrading microbial communities. J Water Process Eng 51:103400. 10.1016/j.jwpe.2022.103400

[CR14] Fakhru’l-Razi A, Pendashteh A, Abdullah LC, Biak DR, Madaeni SS, Abidin ZZ (2009) Review of technologies for oil and gas produced water treatment. J Hazard Mater 170(2–3):530–551. 10.1016/j.jhazmat.2009.05.044 19505758 10.1016/j.jhazmat.2009.05.044

[CR15] Ghosh S, Chakraborty S (2019) Influence of inoculum variation on formation and stability of aerobic granules in oily wastewater treatment. J Environ Manage 248:109239. 10.1016/j.jenvman.2019.07.010 31306929 10.1016/j.jenvman.2019.07.010

[CR16] Igunnu ET, Chen GZ (2012) Produced water treatment technologies. Int J Low-Carbon Technol 9(3):157–177. 10.1093/ijlct/cts049

[CR17] Jepsen KL, Bram MV, Pedersen S, Yang Z (2018) Membrane fouling for produced water treatment: a review study from a process control perspective. Water 10(7):847. 10.3390/w10070847

[CR18] Kebede G, Tafese T, Abda EM, Kamaraj M, Assefa F (2021) Factors influencing the bacterial bioremediation of hydrocarbon contaminants in the soil: Mechanisms and impacts. J Chem 2021:9823362. 10.1155/2021/9823362

[CR19] Kehrmann J, Wessel S, Murali R, Hampel A, Bange FC, Buer J, Mosel F (2016) Principal component analysis of MALDI TOF MS mass spectra separates *M. abscessus* (sensu stricto) from *M. massiliense* isolates. BMC Microbiol 16:24 26926762 10.1186/s12866-016-0636-4 PMC4772520

[CR20] Koster CG, Brul S (2016) MALDI-TOF MS identification and tracking of food spoilers and food-borne pathogens. Curr Opin Food Sci 10:76–84

[CR21] Lee K, Lee SS (2016) *Aneurinibacillus humi* sp. *nov.*, isolated from soil collected in Ukraine. Curr Microbiol 72:139–144. 10.1007/s00284-015-0930-7 26542530 10.1007/s00284-015-0930-7

[CR22] Martina MS, Santosa IC, Carlton DD, Stigler-Granadosc P, Hildenbrandb ZL, Schuga KA (2018) Characterization of bacterial diversity in contaminated groundwater using matrix-assisted laser desorption/ionization time-of-flight mass spectrometry. Sci Total Environ 622–623:1562–1571. 10.1016/j.scitotenv.2017.10.027 10.1016/j.scitotenv.2017.10.027 29054663

[CR23] Olowomofe TO, Oluyege JO, Aderiye BI, Oluwole OA (2019) Degradation of poly aromatic fractions of crude oil and detection of catabolic genes in hydrocarbon-degrading bacteria isolated from Agbabu bitumen sediments in Ondo State. AIMS Microbiol 5(4):308. 10.3934/microbiol.2019.4.308 31915745 10.3934/microbiol.2019.4.308 PMC6946641

[CR24] Palanimanickam A, Sepperumal U (2017) Degradation of l*ambda cyhalothrin* in soil inoculated with *Bacillus cereus* and *Aneurinibacillus migulanus*. J Pure Appl Microbiol 11(4):2017–2021. 10.22207/JPAM.11.4.44

[CR25] Pascale TAM, Rosine YD, Sabine VN, Eric YK, Julien CK (2024) Principal component analysis (PCA) of MALDI-TOF for the identification of waterborne pathogenic bacteria. Microbiol Res J Int 34(11):1–16. 10.9734/mrji/2024/v34i111494

[CR27] Santos IC, Hildenbrand ZL (2016) Schuga KA (2016) Applications of MALDI-TOF MS in environmental microbiology. Analyst 141:2827–2837. 10.1039/C6AN00131A 27072574 10.1039/c6an00131a

[CR28] Wang X, Jiang L, Gai Z, Tao F, Tang H, Xu P (2018) The plasticity of indigenous microbial community in a full-scale heavy oil-produced water treatment plant. J Hazard Mater 358:155–164. 10.1016/j.jhazmat.2018.06.049 29990802 10.1016/j.jhazmat.2018.06.049

[CR29] Zhang Y, Gao J, Li Q, Yang J, Gao Y, Xue J, Li L, Ji Y (2024) Biosurfactant production by *Bacillus cereus* GX7 utilizing organic waste and its application in the remediation of hydrocarbon-contaminated environments. World J Microbiol Biotechnol 40:334. 10.1007/s11274-024-04115-7 39358641 10.1007/s11274-024-04115-7

[CR30] Zhou H, Chen C, Zhou S, Bu K, Li P, Lin X, Zhang C (2021) Performance and microbial community analysis of a bio-contact oxidation reactor during the treatment of low-COD and high-salinity oilfield produced water. Bioresour Technol 335:125267. 10.1016/j.biortech.2021.125267 33992912 10.1016/j.biortech.2021.125267

