# Supplementary material for: Exploring the diversity of produced water bacteria with hydrocarbon-degrading potential using MALDI-TOF MS and multivariate statistical analyses
Source: Biotechnol Lett. 2025 Sep 26;47(5):116. doi: 10.1007/s10529-025-03641-0 (PMC12474702; doi:10.1007/s10529-025-03641-0)
Supplement: Supplementary file 1 — Supplementary file1 (DOCX 414 KB) [file 10529_2025_3641_MOESM1_ESM.docx]

**Supplementary File**

| **MK1** |
| --- |
| **MK4** |
| **MK5** |
| **MK6** |
| **MK7** |
| **MK9-1** |
| 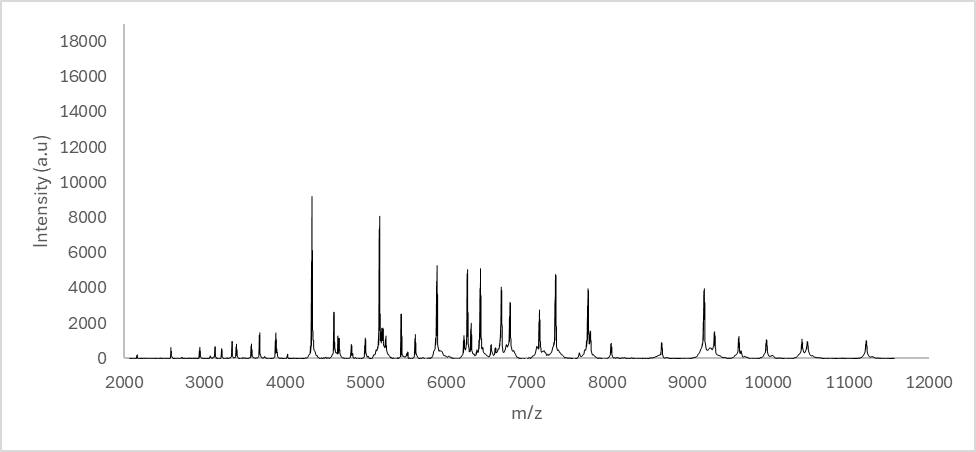  **MK9-2** |
| 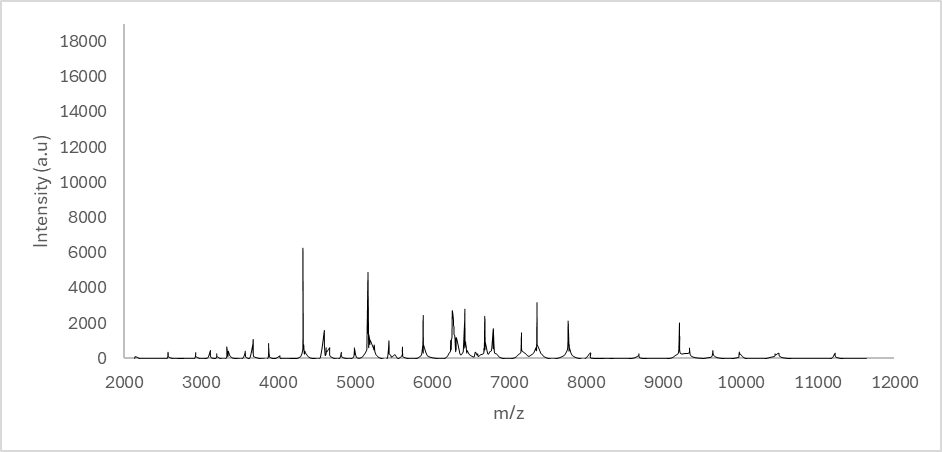  **MK10** |
| **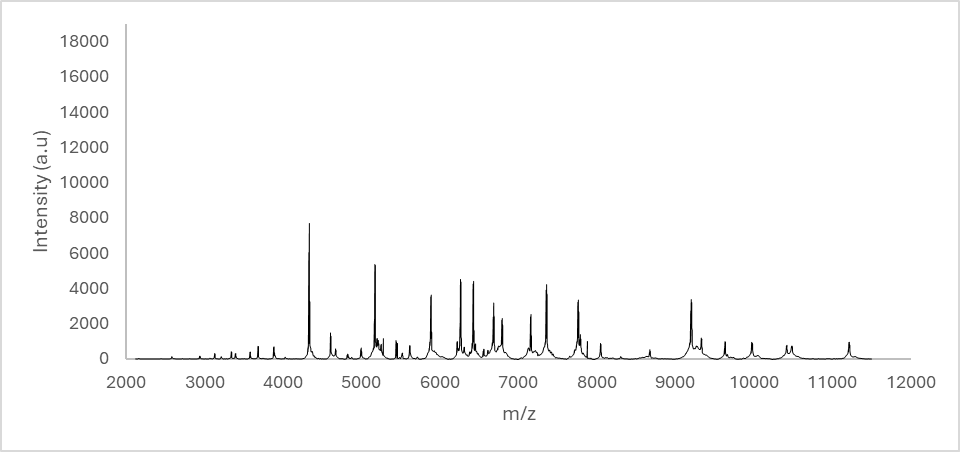**  **MK11-2** |
| **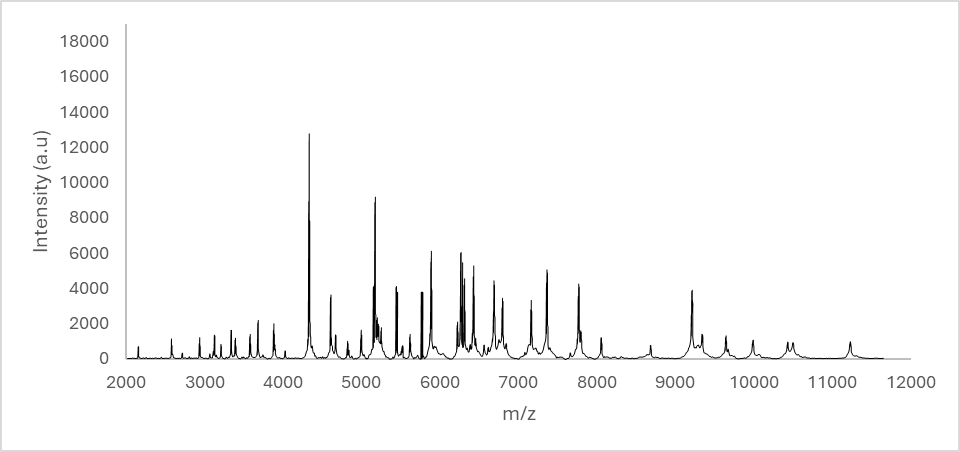**  **MK12** |

**Figure S1.** MALDI-TOF MS protein spectra of B. cereus strains (MK1, MK4, MK5, MK6, MK7, MK9-1, MK9-2, MK10, MK11-2, MK12).
